# Microbial burden and viral exacerbations in a longitudinal multicenter COPD cohort

**DOI:** 10.1186/s12931-020-01340-0

**Published:** 2020-03-30

**Authors:** Jerome Bouquet, David E. Tabor, Jonathan S. Silver, Varsha Nair, Andrey Tovchigrechko, M. Pamela Griffin, Mark T. Esser, Bret R. Sellman, Hong Jin

**Affiliations:** 1 Clinical Pharmacology & Safety Sciences, Biopharmaceuticals R&D, AstraZeneca, South San Francisco, USA; 2 Respiratory Inflammation and Autoimmunity, Biopharmaceuticals R&D, AstraZeneca, Gaithersburg, USA; 3 Data Science and AI, Biopharmaceuticals R&D, AstraZeneca, Gaithersburg, USA; 4 Microbial Sciences, Biopharmaceuticals R&D, AstraZeneca, Gaithersburg, USA

## Abstract

**Background:**

Chronic obstructive pulmonary disease (COPD) is a heterogeneous disease characterized by frequent exacerbation phenotypes independent of disease stage. Increasing evidence shows that the microbiota plays a role in disease progression and severity, but long-term and international multicenter assessment of the variations in viral and bacterial communities as drivers of exacerbations are lacking.

**Methods:**

Two-hundred severe COPD patients from Europe and North America were followed longitudinally for 3 years. We performed nucleic acid detection for 20 respiratory viruses and 16S ribosomal RNA gene sequencing to evaluate the bacterial microbiota in 1179 sputum samples collected at stable, acute exacerbation and follow-up visits.

**Results:**

Similar viral and bacterial taxa were found in patients from the USA compared to Bulgaria and Czech Republic but their microbiome diversity was significantly different (*P* < 0.001) and did not impact exacerbation rates. Virus infection was strongly associated with exacerbation events (*P* < 5E-20). Human rhinovirus (13.1%), coronavirus (5.1%) and influenza virus (3.6%) constitute the top viral pathogens in triggering exacerbation. Moraxella and Haemophilus were 5-fold and 1.6-fold more likely to be the dominating microbiota during an exacerbation event. Presence of Proteobacteria such as Pseudomonas or Staphylococcus amongst others, were associated with exacerbation events (OR > 0.17; *P* < 0.02) but more strongly associated with exacerbation frequency (OR > 0.39; *P* < 4E-10), as confirmed by longitudinal variations and biotyping of the bacterial microbiota, and suggesting a role of the microbiota in sensitizing the lung.

**Conclusions:**

This study highlights bacterial taxa in lung sensitization and viral triggers in COPD exacerbations. It provides a global overview of the diverse targets for drug development and explores new microbiome analysis methods to guide future patient management applications.

## Background

Chronic obstructive pulmonary disease (COPD) is defined by airflow limitation but encompasses several lung diseases. This heterogeneity includes differences in clinical characteristics, source of inflammation, response to therapies and causes of exacerbation [1]. As COPD progresses, exacerbations become more frequent and more severe. Exacerbation rates reflect an independent susceptibility phenotype [2], which could be mediated by host factors [3], environmental factors [4], viral infections and/or the bacterial microbiome [5, 6].

Infections are predominant causes of COPD exacerbations, with approximately half reported to be caused by bacterial infections including non typeable *Haemophilus influenzae* (NTHi), *Moraxella catarrhalis*, *Streptococcus pneumoniae*, or *Pseudomonas aeruginosa*, and the other half by viral infections, primarily human Rhinovirus (HRV), but also Influenza virus, Coronavirus and Respiratory syncytial virus (RSV) to name a few [5, 6]. Bacteria and viruses are also frequently isolated in the airways of stable COPD patients [6–8]. The advent of culture-independent testing has suggested viral persistence [9] and colonization of the lower airways with a resident bacterial microbiota [10], implicating a role for the microbiota in disease pathogenesis, progression and treatment outcome of lung diseases [7].

Studies of the microbiome provide a new framework to understand host-pathogen interactions, which can also yield new markers for patient diagnosis and management. Microbiota diversity is seen as a potential biomarker in cases where a single pathogenic organism reduces community complexity such as in bacterial vaginosis [11], or Crohn’s disease [12]. Lung microbial dysbiosis in COPD is characterized by decreased diversity [10–12], which may contribute to altered immune response to environmental insults [13]. Dysbiosis at the time of COPD exacerbation contributes to increased disease severity [14] and higher 1-year mortality rates [15].

Geography could also be a potential covariate in COPD patients microbiota. The gut microbiota has been shown to be geographically variable [13]. Previous studies in different conntries have evaluated the COPD microbiota [16, 17], but the effect of geographical variations has not yet been evaluated in a single study in relation to disease severity. The present cohort stems from a study on the incidence of viral infections in COPD [5]. Patients were enrolled in Europe and North America and followed up for up to 3 RSV seasons, with scheduled wellness visits and unscheduled illness visits. To further our understanding of COPD exacerbation dynamics, we retrospectively evaluated the sputum bacterial microbiota from a subset of this study. The goals were to identify differences in patients with higher rates of exacerbations, to assess geographical differences in the microbiota between Europe and the USA, and to determine the influence of viral infections on microbiota diversity and the frequency of exacerbations.

## Materials and methods

### Study cohort

**Fig. 1:**
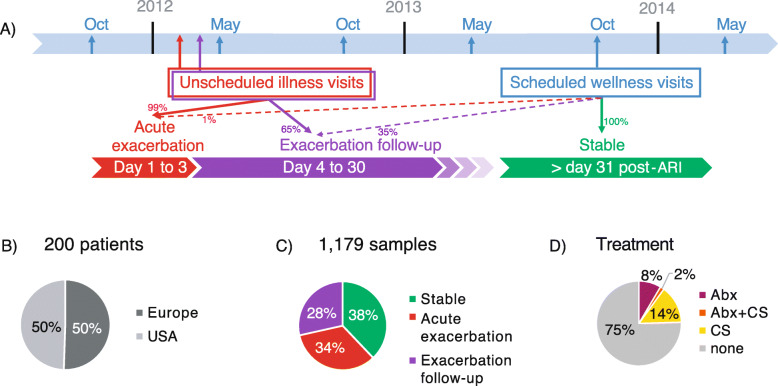
Sampling timeline and composition. (**a**) Timeline from Oct 2011 to May 2014, COPD patients were enrolled and sampled during scheduled wellness visits (blue arrows), and any unscheduled visits (red and purple arrows) within 3 days of an acute exacerbation or exacerbation follow-up visit. Samples were considered stable if collected 31 days post-hospitalization or ARI. Dotted arrows correspond to samples collected at scheduled wellness visits that incidentally corresponded to acute exacerbation events (1.2%) and exacerbation follow-up visits (65%). Piecharts represent the proportion of patients from Europe and USA (**b**), the proportion of samples collected at each disease state (**c**) and the proportion of samples associated with antibiotics (Abx) and inhaled corticosteroids (CS) taken in the past 7 days (**d**)

The patient cohort is part of an observational study on the incidence of acute respiratory illness (ARI) or events leading to the worsening of cardiorespiratory status in COPD (ClinicalTrials.gov, NCT01455402) [13]. The protocol was approved by independent institutional review boards, and all subjects signed written informed consent at enrollment. The study population included 200 adults ≥50 years of age with COPD, recruited at 6 sites in Bulgaria, 5 sites in Czech Republic, and 14 sites across the USA from fall 2011 to spring 2014 (Fig. 1a). Cohort from Bulgaria and Czech Republic showed similar characteristics, and are analyzed jointly as Europe for ease of representation. Subjects had scheduled wellness visits in May and October each year to obtain sputum and clinical data (Fig. 1a). Unscheduled illness visits to collect sputum and clinical data were performed when a subject experienced an ARI or acute exacerbation of COPD symptoms, and during follow-up illness visits. Samples were considered stable if collected at least 30 days from the last day of hospitalization or from the last ARI or acute exacerbation event if it did not require hospitalization. Acute exacerbation samples were collected within 72 h of an event, additional samples were collected 4–30 days after an acute exacerbation event during unscheduled visits (Fig. 1a, c). Patients were characterized with a frequent exacerbator phenotype if they experienced 2 or more exacerbation events per year. No investigational drug was administered in the study. The subject’s physician prescribed and recorded all treatment deemed necessary to provide adequate supportive care. Samples collected 1 to 7 days following treatment were considered treatment associated (Fig. 1d).

### Sputum sample collection

Spontaneous rather than induced sputum collection was possible in this severe COPD study subset following a standardized collection visit [18]. Subjects were asked to gargle with water immediately prior to sputum collection to reduce the number of oral bacteria [19]. Subjects were asked to cough deeply and expectorate into a cup, mixed 1:1 with cold Transport Media and kept at -60 °C or below [20].

### Viral testing

The GenMark respiratory virus panel (GenMark Diagnostics, Inc. Carlsbad, CA) was used to detect common respiratory viruses from all sputum samples. RSV detection was confirmed by RT-PCR as previously described [5]. Additionally, RT-PCR using primers against HRV VP1-VP4 [21] was used to detect human rhinovirus subtypes.

### 16S rRNA gene sequencing

Bacterial genomic DNA was extracted at a single central lab using the Zymobiomics 96 DNA kit (Zymo, California, USA) following manufacturer’s instructions. The V4 hypervariable region of the 16S rRNA gene was PCR amplified [22] and sequenced using Illumina Miseq platform along with negative controls, Zymobiomics Microbial community and DNA standards, and a PhiX library for quality control.

### Bioinformatic analysis

16S rRNA gene sequences were analysed using QIIME2 version 2018.11 [23]. DADA2 software package [24], wrapped in QIIME2, was used for correcting sequences and obtained 9853 annotated sequence variants (ASVs). Taxonomy was assigned using a Naïve Bayes classifier [25, 26] that was trained on the Greengenes database version 13.8 clustered at 99% identity [27] V4 sequences. Alignment was performed with MAFFT [28], masked and used in FastTree [29] to build the phylogenetic tree. Alpha-diversity metrics [30], beta diversity metrics [31], and Principal coordinate analysis (PCoA) were estimated after samples were rarefied to 1000 sequences per sample. Significant features of interest were re-tested using non-rarefying alpha [32] and beta diversity estimates [33]. Analysis of composition (ANCOM) was used for differential taxa abundance calculations [34] on non-rarefied data. ANCOM account for the structure of the data and controls for the false discovery rate. Microbiota profiles were clustered into biotypes using the biotypeR package in R [35]. Longitudinal microbiota variations at stable time points were assessed by calculating the median weighted UniFrac distance [36] for patients with at least 3 stable samples. Patients were categorized into the bottom and top quartile of within-patient stable samples median UniFrac distances, respectively.

### Statistics

Associations of the microbiota and viral components with demographics and clinical data were calculated in R using Fisher’s test for categorical variables, Welsh’s T-test for comparison of means in continuous variables, and chi-squared test to compare expected frequencies. Odds ratio were calculated using questionr package in R [37]. Associations of microbial diversity with demographics and clinical data were calculated with Qiime2’s diversity plugin using grouped and pairwise Kruskal-Wallis test corrected for false discovery rate for analysis of alpha diversity, and PERMANOVA following 999 permutations for analysis of beta diversity distances.

## Results

### Cohort characteristics

The cohort was composed of 200 patients, 101 from Europe (Bulgaria and Czech Republic) and 99 from the USA (Fig. 1 and Table 1). All but 5 patients presented with severe or very severe COPD. Patients from Europe and the USA were matched by age, sex, and GOLD stage. At enrollment, their forced expiratory volume as a percent of predicted (FEV1%) and their comorbidities (congestive heart failure, diabetes, hypertension and malignancy) were similar between Europe and the USA. The USA patients had significantly higher smoking history in pack-years and longer COPD duration. The number of exacerbations per year and the number of frequent exacerbator phenotype, defined as 2 or more exacerbations/year, was higher in the USA compared to Europe, but not statistically significant (Table 1). A total of 1179 sputum samples were collected from these patients over 3 years, with similar proportions of samples collected at acute exacerbation in Europe (33.3%) and in the USA (33.9%) (Supplementary Table 1). Approximately 11 and 16% of samples were associated with antibiotic and corticosteroid treatment, respectively. Significantly more samples collected in the USA were associated with antibiotic treatment (Supplementary Table 1).

**Table 1:** Patient demographics and major clinical history

Age at Basine, years Patient characteristics*	Europe^**a**^ (***n*** = 101)	USA^**b**^ (***n*** = 99)	***P***-value
**Female**	37 (36.7%)	33 (33.3%)	0.6
**Age at Baseline, years**	65 [50–81]	66 [51–93]	0.2
**Smoking status**			***0.008***
Current	30 (29.7%)	26 (26.8%)	
Former	54 (53.5%)	67 (69%)	
Non-smoker	17 (16.8%)	4 (4.1%)	
**Smoking history, pack-years**	31 [0–90]	51 [0–128]	***1.0E-06***
**Years of COPD**	9 [1–32]	11 [1–34]	***0.02***
**Years of severe COPD**	4 [1–15]	7 [1–33]	***5.0E-04***
**GOLD**^**c**^**stage**			0.9
Mild	1 (0.9%)	0 (0%)	
Moderate	2 (2%)	2 (2%)	
Severe	69 (68.3%)	66 (68.8%)	
Very Severe	29 (28.7%)	28 (29.2%)	
**FEV1**^**d**^	39 [17–85]	37 [13–71]	0.1
**Frequent exacerbator**^**e**^	18 (17.8%)	29 (29.3%)	0.06
**Exacerbations per year**	1.1 [0–11.8]	1.7 [0–12.1]	0.05
**Congestive heart failure**	28 (27.7%)	16 (16.2%)	0.06
**Diabetes**	25 (24.8%)	29 (29.9%)	0.4
**Hypertension**	61 (60.4%)	70 (72.1%)	0.08
**Malignancy**	2 (2%)	5 (5.1%)	0.2

* Categorical data presented as number (proportion), and continuous variable as mean [range]

^a^ Patients recruited at 11 sites in Bulgaria and Czech Republic

^b^ Patients recruited at 14 sites in Alabama, Arizona, Georgia, Iowa, Nevada, New York, Ohio, Pennsylvania, Tennessee, Texas, Wisconsin

^c^ Global Initiative for Chronic Obstructive Lung Disease

^d^ Forced Expiratory Volume in 1 s, expressed in % of predicted based on height and weight

^e^ ≥ 2 exacerbations/ year

### Overview of 16S microbiota and viral detection in COPD sputum

**Fig. 2:**
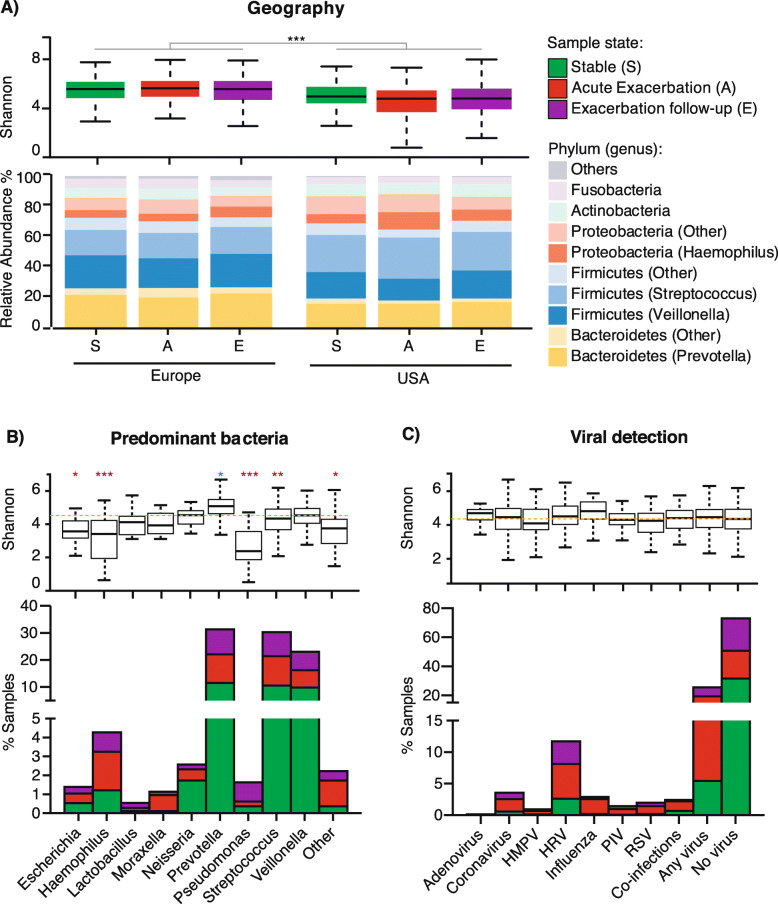
Microbiota composition and diversity is associated with geography, not with acute exacerbation or viral infections. (**a**) Taxonomic barplot of major bacterial phyla and genera in samples grouped by geography and disease state, and their respective Shannon diversity index represented as boxplots with interquantile range whiskers. (**b**) Percentage of samples with microbiota predominant with nine most common COPD bacterial taxa, and (**c**) percentage of samples positive for 7 most common respiratory viruses are plotted at stable (green), acute exacerbations (red) and follow-up visits (purple), and their respective Shannon diversity index. * *P* < 0.01, **P < 0.001, *** *P* < 0.0001, lower and higher statistical significant diversity compared to the average are noted in red and blue, respectively. HMPV, Human Metapneumovirus

Analysis of bacterial taxa in 1179 sputum samples shows phyla commonly observed in the lung microbiota, with Firmicutes, Proteobacteria and Bacteroidetes representing a majority (> 80%) of the phyla identified (Fig. 2a). Prevotella, Veillonella, Streptococcus and Haemophilus represented the most prevalent (> 65%) bacterial genera (Fig. 2a). Samples from European patients had more Bacteroidetes and less Proteobacteria overall than samples from the USA patients. Within the phylum Firmicutes, the USA patients had more Streptococcus than Veillonella compared to European patients (Fig. 2a). Microbiota predominant with Prevotella, Streptococcus and Veillonella were found in a majority of samples (1012/1179; 85.8%) (Fig. 2b). Prevotella, Streptococcus and Veillonella represented the majority of the microbiota in samples collected at stable states, Haemophilus and Moraxella were predominant in acute exacerbation samples, and Pseudomonas was prevalent in exacerbation follow-up samples (Fig. 2b).

Viral testing on 1179 sputum samples showed that 25.6% of sputum samples were positive for at least one virus, with HRV found in the largest proportion of samples (13.6%), followed by Coronavirus (5.1%) and Influenza virus (3.6%) (Fig. 2c). All viruses, except for adenoviruses, were detected more frequently at acute exacerbation (14.4%), than at follow-up visits (7.2%) or stable (5.6%) (Fig. 2c). There were no significant differences in viral incidence between Europe and the USA, with the exception of *Coronavirus HKU1*, *Influenza B virus* and RSV A (Supplementary Table 1).

### Microbiota diversity

Alpha diversity metrics such as the number of observed ASV, Shannon evenness, or Faith phylogenetic diversity (PD) indexes, represent the mean number of taxons in a sample, sometimes weighted by the phylogenetic relatedness. Overall, samples from the USA patients had significantly lower shannon diversity index than those from Europe (Fig. 2a), even when corrected for antibiotic use or clinical sites (Supplementary Fig. 1 and 2). Years of severe COPD and smoking pack-years were associated with differences in microbiota diversity but not as significantly as geography (Supplementary Table 2). No significant differences in the number of observed ASVs and in Faith PD index were observed at exacerbation compared to stable samples (Supplementary Table 2). Prevotella-predominant microbiota showed the highest alpha diversity, while samples predominant with Escherichia, Haemophilus, and Pseudomonas had the lowest alpha diversity (Fig. 2b). There were no significant differences in microbial diversity between samples infected or not with a virus, or between samples infected with different viruses (Fig. 2c). Beta diversity are metrics such as weighted UniFrac and robust Aitchison PCA used for comparing microbiota communities resulting in distance matrices. Here, principal coordinate visualization of weighted UniFrac distance supports the above conclusions, showing some differences in geography and predominant bacteria but not affected by the type of viral infection (Supplementary Fig. 3). These results were also supported by testing non-rarefied alpha and beta-diversity estimates, with geography (*P* < 0.044) and dominant bacterial genus (*P* < 0.001), but not viruses (*P* > 0.094) being associated with significant changes in diversity (data not shown).

### Odds ratio of exacerbation

**Fig. 3:**
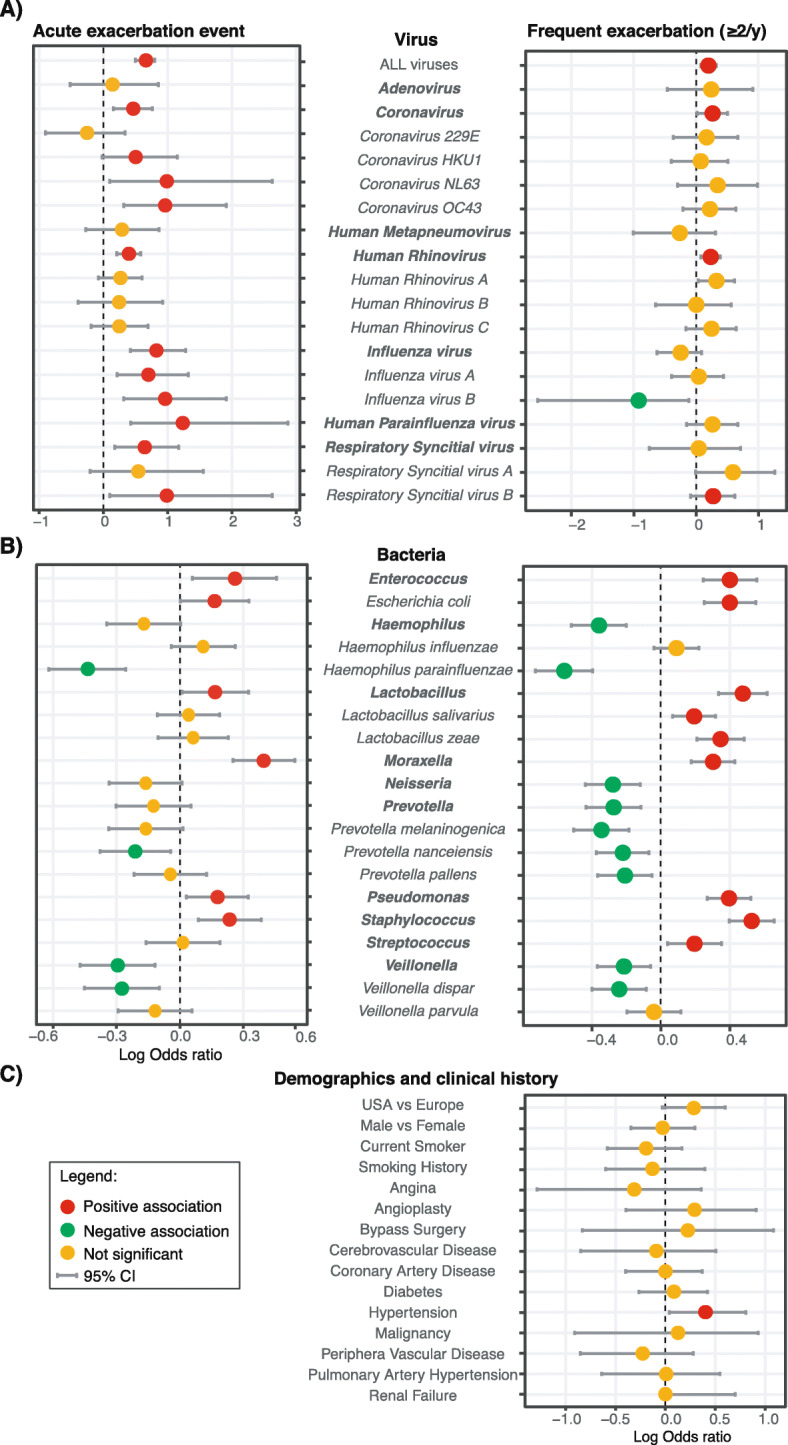
Risk factors of COPD exacerbations. Adjusted odds ratio of (**a**) viral infections (**b**) bacterial abundance (top/bottom quartile) and (**c**) demographics and clinical history features to be associated with acute exacerbation events or patients with frequent exacerbations (≥2 events/ year). Significance are presented in red (positive association) and green (negative association). Orange dots represent non-significant odds ratio. Horizontal bars represent the 95% confidence interval. Genera names are in bold and species italicized

The odds ratio of an acute exacerbation event and frequent exacerbations (≥2 events/ year) was calculated for demographic and clinical data, viral infections and abundance of certain bacterial taxa in the lung microbiota (Fig. 3). Viral infections were more strongly associated with an exacerbation event than with frequent exacerbations. Parainfluenzaviruses (PIV), *Influenza B virus* and RSV B had the highest odds ratio of an exacerbation event (Fig. 3a). Interestingly, *Influenza B virus* was negatively correlated with frequent exacerbations, as it was only detected in patients that exacerbated infrequently.

Bacteria were more strongly associated with exacerbation frequency than with exacerbation events. Presence or higher abundance of Enterococcus, Lactobacillus, Moraxella, Pseudomonas, Staphylococcus and Streptococcus was correlated with frequent exacerbations. Neisseria, Prevotella and Veillonella were significantly associated with a lower exacerbation frequency (Fig. 3b). Interestingly, top quartile abundance odds ratio of the genus Haemophilus was not associated with higher exacerbation rate. This effect, however, seemed mediated by *H.parainfluenzae*. We noted that differential abundance of only 2 bacterial taxa were significantly associated with exacerbation events, while 19 taxa were associated with exacerbation frequency (Supplementary Fig. 4), with high *E.coli*, Lactobacillus, and Staphylococcus in stable samples as potential predictors of frequent exacerbation (Supplementary Fig. 5).

Patients with hypertension had a significantly higher odds ratio of being frequent COPD exacerbators (Fig. 3c). Other comorbidities did not significantly influence exacerbation frequency.

### Longitudinal variations of stable state sputum microbiota and viruses

**Fig. 4:**
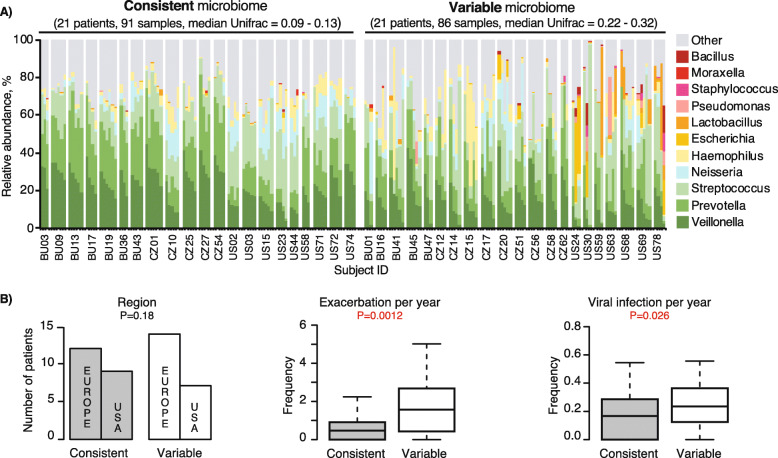
Longitudinal variations of the COPD sputum microbiota at stable state is associated with higher disease burden. **a** Longitudinal Taxa barplot at Genus level of all 42 patients representing the top (variable) and bottom (consistent) quartile of median weighted UniFrac distances at stable states. **b** Proportions of patients with low or high longitudinal microbiota variability associated with geography, and boxplots with interquantile range whiskers for the frequency of exacerbations, and of viral infections

We quantified temporal variability of the sputum microbiota at stable state within individual subjects. For 84 patients with more than 3 longitudinal stable samples, we calculated their median weighted UniFrac distances and categorize the top and bottom quartile patients into consistent and variable microbiota over time (Fig. 4a). Patients with a more variable sputum microbiota (median weighted UniFrac > 0.22) were more likely to have a higher relative abundance of Bacillus, Escherichia, Lactobacillus, Moraxella, and Staphylococcus (Fig. 4 and Supplementary Fig. 6). Microbiota variability in seemingly stable disease state were associated with higher exacerbation frequency and frequent viral infections (Fig. 4b).

**Fig. 5:**
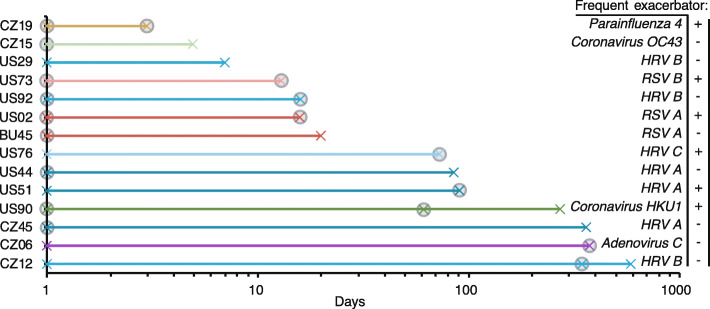
Timeline of consecutive viral detection. Longitudinal viral detection in 14 patients as indicated by a cross and lines are colored according to virus species, strain or subtype. Exacerbation events are indicated by a filled circle, and frequent exacerbator phenotypes are indicated by plus or minus signs

Longitudinal sampling also enabled the assessment of recurrent viral infections by the same viral species, strain or subtype in consecutive samples. Viruses were detected in 2 to 3 consecutive samples from 14 patients (Fig. 5). The time beween 2 consecutive samples ranged from 3 days to 1 year. Viruses that were detected within 2–3 weeks of sampling corresponded most probably to a typical single infection, and these included the detection of *Coronavirus OC43*, HRV A, PIV4, RSV A and B. Viruses that were detected in the same patient over 60 days apart may correspond to chronic or recurrent infections, with *Adenovirus C*, *Coronavirus HKU1*, HRV A, HRV B and HRV C detected. Eighteen out of 30 (60%) samples from these 14 patients corresponded to acute exacerbation events, and 6/14 (42%) patients were frequent exacerbators (Fig. 5).

### Biotyping of COPD sputum microbiota

**Fig. 6:**
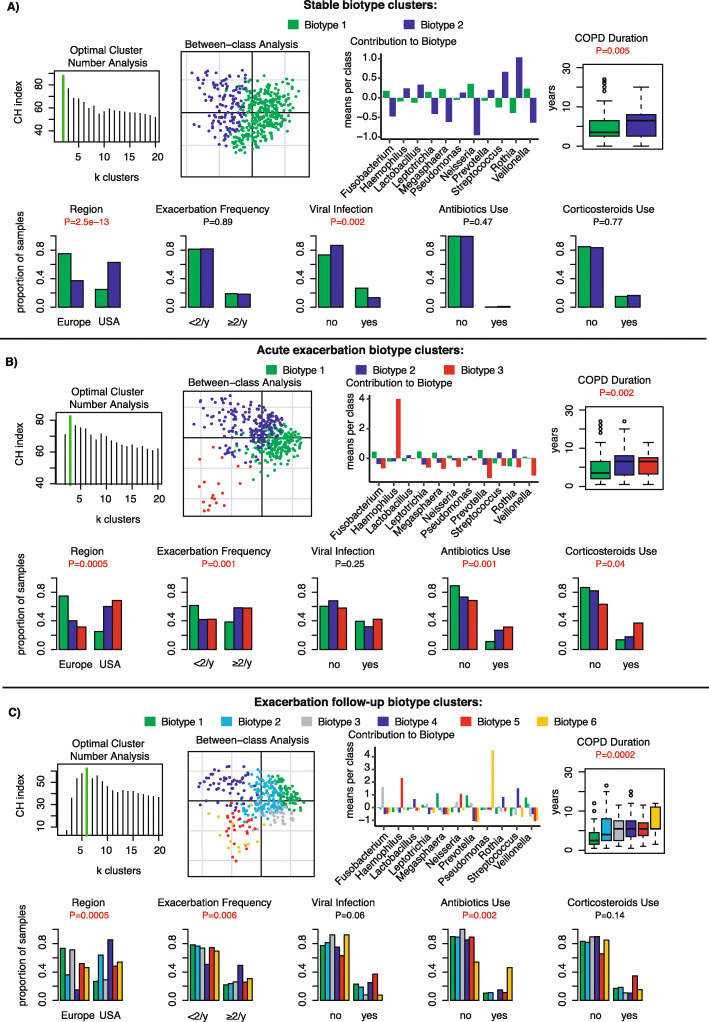
Biotyping clusters of COPD sputum microbiota at stable, acute exacerbation and follow-up visits. For each state, optimal cluster number analysis using the Calinski-Harabasz index, scatter diagram of samples clustered by between-class principal component analysis, contribution of major bacterial genera in means per class and proportion of samples belonging to each cluster plotted by region, frequency of exacerbation, frequency of of viral exacerbations, antibiotics use, corticosteroids use and CODP duration are presented at stable (**a**), acute exacerbated (**b**) and exacerbated follow-up visits (**c**)

We sought to define common microbiota clusters and their association with clinical characteristics. Microbiota in stable samples could be separated into 2 biotypes, as indicated by the highest Calinski-Harabasz (CH) index following iterative partitioning-around-medoids clustering analysis over the Jensen-Shannon distance. Between-class analysis showed 2 major clusters, biotype 1 represented by Neisseria and Veillonella, and biotype 2 represented by Streptococcus and Rothia (Fig. 6a). Samples with high relative abundance of Streptococcus/Rothia (biotype 2) were found in greater proportion in USA patients, which was associated with longer history of COPD and less frequent detection of viruses at stable visits (Fig. 6a). Samples at acute exacerbation visits could be separated into 3 biotypes, characterized by a high relative abundance of either Prevotella (biotype 1), Streptococcus (biotype 2), or Haemophilus (biotype 3) (Fig. 6b). Streptococcus and Haemophilus were found in a majority of USA samples, and associated with longer COPD duration, higher exacerbation frequency, antibiotics and corticosteroid use, but did not significantly correlate with higher viral infections (Fig. 6b). Exacerbation follow-up samples were more diverse and could be optimally clustered into 6 biotypes. Biotype 6 was characterized by a high relative abundance of Pseudomonas and significantly associated with longer COPD duration and antibiotic use. Biotype 4 was characterized by a high relative abundance of Streptococcus or Rothia and was significantly associated with higher exacerbation frequency. Other biotypes were not significantly associated with clinical characteristics (Fig. 6c). Biotyping shows that microbiota profile diversity is dynamically dependent on disease state.

## Discussion

Understanding of the presence and role of both bacterial and viral pathogens over time in the heterogeneous and dynamic COPD disease [38] is needed for patient treatment and management. The characterization of the 16S rRNA gene microbiota and respiratory viruses from a longitudinal and international cohort of severe COPD patients described in this study provides the largest survey to date on their complex associations with geography, exacerbation frequency and other demographic and clinical history.

COPD patients are particularly susceptible to respiratory infections [6]. HRV was identified as the most prominent agent in respiratory tract infections in this cohort of COPD patients. Viral characterization is of particular importance as few reports exist on the diversity of respiratory viral agents in COPD [6] and viral diversity should be accounted for when designing new treatments. In particular, the newly described HRV C was detected in 23 of 11,179 samples (21 of 200 patients) in the present cohort, which was reported only once previously [39].

We detected viruses in 15% of stable samples. Asymptomatic viral infections in COPD patients are common [40]. However, the role of these asymptomatic infections in disease progression is unclear. Most respiratory viruses tested here showed highly significant positive odds ratio with exacerbation events, but lower significance in regards to exacerbation frequency. This may indicate that viral infections alone do not sensitize the lung to repeat exacerbations as much as the bacterial microbiota. Interestingly, similar trends have been shown in asthma where respiratory viral infections in early life resulted in microbiome changes and hypersensitivity predisposition that can lead to asthma [41, 42].

Repeat viral detection were more frequent in patients with frequent exacerbator phenotype, but the number was small, and contradicting reports exist on the repeated detection of a single virus in COPD [9]. Further complete viral genomic characterization will be needed to understand the nature of viral infection.

With the advent of culture-independent techniques, it appears that all microbiomes harbor potential bacterial pathogens as characterized here and elsewhere [14], but that only a portion of them will develop exacerbation-prone phenotypes, a phenotype that appears independent of disease GOLD stage yet linked to microbiota diversity [13]. It was previously observed that microbiota diversity in the COPD lung correlated with disease severity but not disease state [43]. Here, microbiota diversity alone was not correlated with frequent exacerbations, but was highly correlated with certain bacterial taxa dominating the microbiota. Microbiota predominant with Escherichia, Pseudomonas or Streptococcus, showed significantly lower alpha diversity and significant positive odds ratio with the frequent exacerbation phenotype, suggesting a role of the microbiota in sensitizing the COPD lung to acute exacerbations. This study was limited in disease severity metrics with the exception of baseline evaluation and sampling of events. Longitudinal monitoring of symptoms scales would help to better understand the relation of certain bacteria to not only exacerbation frequency, but also the symptom severity and COPD progression.

The use of biotyping has been seldom used in respiratory microbiota research [44] and not yet explored in COPD. A complex resident bacterial community could be identified in all COPD sputum samples and categorized into 2, 3 and 6 biotypes at stable, acute exacerbation and exacerbation follow-up visits, respectively. Biotype 1 at stable state was associated with higher viral infections, while biotypes 2 and 3 at acute exacerbation were associated with high exacerbation frequency. These findings are interesting because they mirror another study showing the partitioning of COPD exacerbation samples into 3 cytokine profile clusters [3] with associations to specific ratios of Proteobacteria, Firmicutes and Bacteroidetes that highlighted the heterogeneity of exacerbation profiles in COPD patients. During exacerbation follow-up visits, biotype 6 with a high relative abundance of Pseudomonas was found over-represented in samples associated with antibiotics use. Antibiotic treatment inadequacy is the cause for secondary infection or the emergence of multi-drug resistant *P.aeruginosa* [45]*.* New targeted treatments, such as monoclonal antibodies, could be useful in such settings [46].

The principal novelty of this study cohort was the long term patient follow-up. We were able to collect several sputum samples per patient at stable state over the course of 3 years and studied the COPD microbiota longitudinally. The lung microbiome is inherently variable, shaped by a process of inhalation and elimination [47]. The lung microbiome is also personal, with large inter-patient variability [48]. Previously, it was shown that microbial dysbiosis from stable to exacerbated state correlated with greater exacerbation severity [14]. Here, patients with greater microbiota variability at stable state correlated with higher exacerbation frequency. Proteobacteria such as Pseudomonas and Moraxella were more abundant in patients with more variable microbiota at stable state. Interestingly, *P.aeruginosa* and *M.cattharalis* are prominent causes of exacerbations [6], but their role in stable disease is less clear [49]. It was previously shown that chronic colonization with *P. aeruginosa* occurs more frequently in more severe COPD patients [50] and that *M.catarrhalis* asymptomatic colonization was associated with a greater frequency of a sputum IgA response than exacerbation [51]. Our results suggest that dysbiotic burden at stable state by Pseudomonas, Moraxella and others might sensitize the lung to further exacerbations and viral infections. Pseudomonas and Moraxella, like many opportunistic Proteobacteria, are pro-inflammatory [52, 53]. Imbalanced inflammation can improve *P.aeruginosa*’s fitness [52], allow the acquisition of new *M.cattharalis* strains [53], leading to exacerbation and possibly infections from other pathogens in a coupled cycle of inflammation and dysbiosis [47]. Microbiology clinical testing in COPD patients is most often performed at exacerbation or follow-up visits. Patients might benefit from clinical monitoring of these bacteria at stable state to assess their presence and/or growth which could lead to potential future exacerbations.

We also noted geographical differences in COPD lung microbiota. Geographical differences in gut microbiota have previously been noted [35], but not yet in the lung. There were significant differences in alpha and beta diversity between the USA and Europe, but not within countries or sites. Microbiota diversity in the USA was lower and although frequent exacerbator phenotypes were more common than in Europe, the difference was not significant. USA patients tended to have samples with high relative abundance of Streptococcus (biotype 2) and Haemophilus (biotype 3) associated with the frequent exacerbator phenotype. *S.pneumoniae* and *H.influenzae* are commonly associated with exacerbations [54], and should also be considered as potential risk factors in the frequent exacerbator phenotype. Significantly more samples collected in the USA were associated with antibiotics use, but this alone did not explain differences in diversity. This observational study included a variety of standard-of-care medications, doses and timings precluding precise treatment effect modeling on the microbiota. Clinical trials exploring current and novel treatment modalities will lead to better patient management and antibiotics stewardship as reviewed elsewhere [55].

Predominance of Haemophilus was over-represented in acute exacerbation samples (Figs. 2 and 6), as noted in previous studies [6, 14, 17, 43, 56]. However, interestingly, using odds ratio (Fig. 3) or ANCOM (Suppplementary Fig. 4a) over Haemophilus abundance, *H.influenzae* was high but not significantly associated with acute exacerbation event, whereas *H.parainfluenzae* was significantly higher at stable state. Differences in Haemophilus abundance compared to other studies might be due to the type of sputum collected, transportation media, extraction protocol or an effect of the sample size. Although *H.parainfluenzae* can be the cause of respiratory infection in healthy subjects, it has not been associated with exacerbation in COPD [56]. *H.parainfluenzae* could compete for niche resources leading to overgrowth of the more pathogenic *H.influenzae*. Previous work has shown that *H.influenzae* competes with *S.pneumoniae* [57] and that patients colonized by NTHi and acquiring HRV have more frequent and severe exacerbations [58]. Here, Streptococcus species could not be resolved using 16S rRNA V4 region, and although speciation of Haemophilus was attempted, further validation using targeted PCR or whole genome sequencing will be necessary to ensure correct discrimination. To note, constant improvements in 16S databases can also affects taxonomic resolution. SILVA database [59] version 132 updated in 2017 classified reads into more genera (*n* = 562) compared to Greengenes version 13.8 updated in 2013 (*n* = 395). However, bacterial taxa discussed in this study showed less than 1% variations in read classification between the 2 database classifiers (data not shown), and conclusions were unchanged. Speciations and typing of bacteria and viruses are critical to understanding their pathogenicity and complex relationships. Greater taxonomic resolution will be achieved using updated databases and more comprehensive techniques like shotgun metagenomics.

## Conclusion

In summary, our study provides a broad survey of viruses and bacteria colonizing severe COPD patients, providing clinicians with potential targets for clinical testing and patient treatment. It demonstrates that viral infections are strongly associated with acute exacerbation events, and that particular components of the microbiota are associated with higher exacerbation frequency. Geographic and longitudinal differences in the lung COPD microbiota exist and were correlated with exacerbation outcomes. Stable state longitudinal microbiota monitoring and biotyping could lead to the identification of potential biomarkers indicative of future exacerbations from bacterial sensitization. Comprehensive microbiota profiling and respiratory viral detection will be useful in the development of anti-microbial agents for therapeutic intervention or for better patient management.

## Supplementary information

**Additional file 1 : Table S1**. Sample characteristics. **Table S2**. Statistical significance of Alpha and Beta microbiota diversity metrics against demographic and clinical variables using Qiime2’s diversity plugin. **Figure S1.** Shannon diversity following antibiotic treatment in Europe and USA patients. **Figure S2.** Microbiota composition and diversity across study sites. **Figure S3**. Principal coordinate analysis of weighted UniFrac distances of the sputum microbiota colored by (A) geography and sample type, (B) most predominant bacterial taxa, and (C) viral infection. **Figure S4**. Bacteria associated with COPD exacerbation or frequency of exacerbation. (A) Abundance of Moraxella and *H.parainfluenzae* identified by analysis of composition (ANCOM) of microbiota between stable and exacerbated samples. (B) Cladogram of bacterial taxa identified by ANCOM comparing Frequent (> 2 exacerbation event/ year) to Infrequent exacerbator. **Figure S5.** Adjusted odds ratio of bacterial abundance (top/bottom quartile) in stable samples only to be associated with frequent exacerbations. **Figure S6**. Cladogram of 55 Differentially abundant bacterial taxa identified by ANCOM comparing consistent to variable longitudinal microbiota patient profiles at stable state.

## Data Availability

Data underlying the findings described in this manuscript may be obtained in accordance with AstraZeneca’s data sharing policy described at https://astrazenecagrouptrials.pharmacm.com/ST/Submission/Disclosure

## Abbreviations

ANCOM: Analysis of composition
ARI: Acute respiratory illness
ASV: Annotated sequence variants
CH: Calinski-Harabasz index
COPD: Chronic obstructive pulmonary disease
FEV1%: Forced expiratory volume in 1 s as a percent of predicted
GOLD: Global initiative for chronic obstructive lung disease
HRV: Human rhinovirus
NTHi: Non typeable *Haemophilus influenzae*
PIV: Parainfluenzavirus
PD: Phylogenetic diversity
RSV: Respiratory syncytial virus
