# Supplementary material for: Microbial burden and viral exacerbations in a longitudinal multicenter COPD cohort
Source: Respir Res. 2020 Mar 30;21:77. doi: 10.1186/s12931-020-01340-0 (PMC7104712; doi:10.1186/s12931-020-01340-0)
Supplement: Supplementary file 1 — Additional file 1 : Table S1. Sample characteristics. Table S2. Statistical significance of Alpha and Beta microbiota diversity metrics against demographic and clinical variables using Qiime2’s diversity plugin. Figure S1. Shannon diversity following antibiotic treatment in Europe and USA patients. Figure S2. Microbiota composition and diversity across study sites. Figure S3. Principal coordinate analysis of weighted UniFrac distances of the sputum microbiota colored by (A) geography and sample type, (B) most predominant bacterial taxa, and (C) viral infection. Figure S4. Bacteria associated with COPD exacerbation or frequency of exacerbation. (A) Abundance of Moraxella and H.parainfluenzae identified by analysis of composition (ANCOM) of microbiota between stable and exacerbated samples. (B) Cladogram of bacterial taxa identified by ANCOM comparing Frequent (> 2 exacerbation event/ year) to Infrequent exacerbator. Figure S5. Adjusted odds ratio of bacterial abundance (top/bottom quartile) in stable samples only to be associated with frequent exacerbations. Figure S6. Cladogram of 55 Differentially abundant bacterial taxa identified by ANCOM comparing consistent to variable longitudinal microbiota patient profiles at stable state. [file 12931_2020_1340_MOESM1_ESM.pdf]

Supplementary Table 1 – Sputum collection characteristics by COPD disease state, association with treatment and viral detection

Sample characteristics*	Europe (n=669)	USA (n=510)	P-value
Disease state			
Stable[a]	290 (43.3%)	157 (30.8%)	***1.E-05***
Acute exacerbation[b]	223 (33.3%)	173 (33.9%)	0.8
Exacerbation follow-up[c]	156 (23.3%)	180 (35.3%)	***6.E-06***
Treatment at time of sample collection[d]			
Antibiotics	20 (3%)	98 (19.2%)	***3.E-20***
Inhaled corticosteroids	121 (18.1%)	68 (13.9%)	0.06
Viral detection[e]			
Any virus	167 (25%)	135 (26.7%)	0.6
Adenovirus	8 (1.2%)	1 (0.2%)	0.05
Adenovirus BE	2 (0.3%)	0 (0%)	0.2
Adenovirus C	6 (0.9%)	1 (0.2%)	0.1
Coronavirus	30 (4.5%)	30 (5.9%)	0.3
Coronavirus 229E	8 (1.2%)	7 (1.4%)	0.8
Coronavirus HKU1	6 (0.9%)	14 (2.7%)	***0.01***
Coronavirus NL63	7 (1.0%)	3 (0.6%)	0.4
Coronavirus OC43	10 (1.5%)	11 (2.2%)	0.4
Human Metapneumovirus	9 (1.4%)	6 (1.2%)	0.8
Human Rhinovirus (HRV)	88 (13.3%)	72 (14.5%)	0.5
HRV A	20 (3%)	22 (4.3%)	0.2
HRV B	6 (0.9%)	7 (1.4%)	0.4
HRV C	16 (2.4%)	7 (1.4%)	0.2
Influenza virus	29 (4.3%)	14 (2.7%)	0.1
Influenza virus A	13 (1.9%)	11 (2.2%)	0.8
Influenza virus A H1	5 (0.7%)	5 (1.0%)	0.7
Influenza virus A H3	7 (1.0%)	6 (1.2%)	0.8
Influenza virus B	16 (2%)	3 (0.6%)	***0.02***
Parainfluenza virus (PIV)	11 (1.6%)	11 (2.2%)	0.5
PIV 1	0 (0%)	2 (0.4%)	0.1
PIV 2	1 (0.1%)	3 (0.6%)	0.2
PIV 3	3 (0.4%)	5 (1.0%)	0.3
PIV 4	7 (1.0%)	1 (0.2%)	0.08
Respiratory Syncytial virus (RSV)	13 (1.9%)	16 (3.3%)	0.2
RSV A	2 (0.3%)	7 (1.4%)	***0.04***
RSV B	4 (0.6%)	7 (1.4%)	0.2

* Categorical data presented as number (proportion)

a Baseline or >1month following exacerbation event

b Within 3 days of exacerbation onset

c Three days to 1 month following exacerbation event

d Sample collected 1 to 7 days following treatment

e Genmark Respiratory viral panel and in-house RT-PCR (see Methods)

Supplementary Table 2 - Statistical significance of Alpha and Beta microbiome diversity metrics against demographic and clinical variables using Qiime2's diversity plugin

	**Alpha diversity**[a]				**Beta diversity**[b]	
	observed OTUs	Shannon	Evenness	Faith PD	Unweighted Unifrac	Weighted Unifrac
Demographic						
USA vs Europe	5.E-16	1.E-27	3.E-28	4.E-18	1.E-03	1.E-03
Sex	0.07	0.36	0.94	0.06	1.E-03	1.E-03
Smoking Status	0.02	8.E-03	0.03	1.E-03	1.E-03	6.E-03
Pack-years*	1.E-06	9.E-08	2.E-05	9.E-06	1.E-03	1.E-03
Subject ID	7.E-50	1.E-41	6.E-28	1.E-49	1.E-03	1.E-03
Disease severity						
GOLD Stage	3.E-06	9.E-05	0.01	3.E-05	1.E-03	0.02
Years of severe COPD*	1.E-04	2.E-07	1.E-07	3.E-05	1.E-03	1.E-03
Exacerbation event	0.14	1.E-02	3.E-03	0.44	1.E-03	1.E-03
Frequent Exacerbation (>=2/year)	0.12	0.02	0.02	0.07	1.E-03	1.E-03
Comorbidities						
Diabetes	0.89	0.60	0.18	0.51	1.E-03	2.E-03
Malignancy	3.E-07	7.E-08	5.E-05	5.E-08	1.E-03	1.E-03
Pulmonary Artery Hypertension	0.02	2.E-05	1.E-07	0.11	1.E-03	1.E-03
Most dominant Bacteria						
Escherichia	2.E-05	8.E-05	4.E-04	2.E-04	1.E-03	1.E-03
Haemophilus	1.E-08	1.E-11	4.E-12	2.E-07	1.E-03	1.E-03
Lactobacillus	0.19	0.18	0.68	0.09	2.E-03	1.E-03
Moraxella	0.9	0.02	2.E-05	0.98	0.17	1.E-03
Neisseria	0.44	0.59	0.75	0.53	2.E-03	1.E-03
Other	3.E-04	7.E-05	6.E-05	0.01	1.E-03	1.E-03
Prevotella	3.E-27	1.E-47	1.E-50	1.E-23	1.E-03	1.E-03
Pseudomonas	4.E-07	1.E-08	8.E-09	3.E-05	1.E-03	1.E-03
Streptococcus	1.E-03	2.E-08	1.E-08	3.E-03	1.E-03	1.E-03
Veillonella	0.25	0.19	0.08	0.11	1.E-03	1.E-03
Viral infection						
Any virus	0.82	0.48	0.16	0.98	0.16	0.08
Adenovirus	0.39	0.56	0.91	0.27	0.45	0.92
Coronavirus	0.15	0.35	0.41	0.08	0.16	0.25
HMPV	0.86	0.73	0.46	0.82	0.50	0.04
HRV	0.42	0.99	0.57	0.25	0.31	0.23
Influenza	0.13	0.04	0.06	0.14	0.05	0.22
PIV	0.82	0.48	0.22	0.72	0.09	0.03
RSV	0.12	0.02	0.01	0.24	0.17	0.01
Frequent Viral Infections (>=2/year)	0.64	0.30	0.29	0.86	1.E-03	0.01
Treatment						
Antibiotics	0.03	3.E-03	4.E-04	0.07	1.E-03	1.E-03
Inhaled Corticosteroids	3.E-03	9.E-03	0.04	2.E-03	1.E-03	4.E-03

*top vs bottom quartile

[a]Alpha diversity significantce calculated as FDR-corrected P-value from grouped and pairwise Kruskal-Wallis test for analysis of diversity corrected for false discovery rate.

[b]Beta diversity significance calculated as FDR-corrected P-values using PERMANOVA following 999 permutations

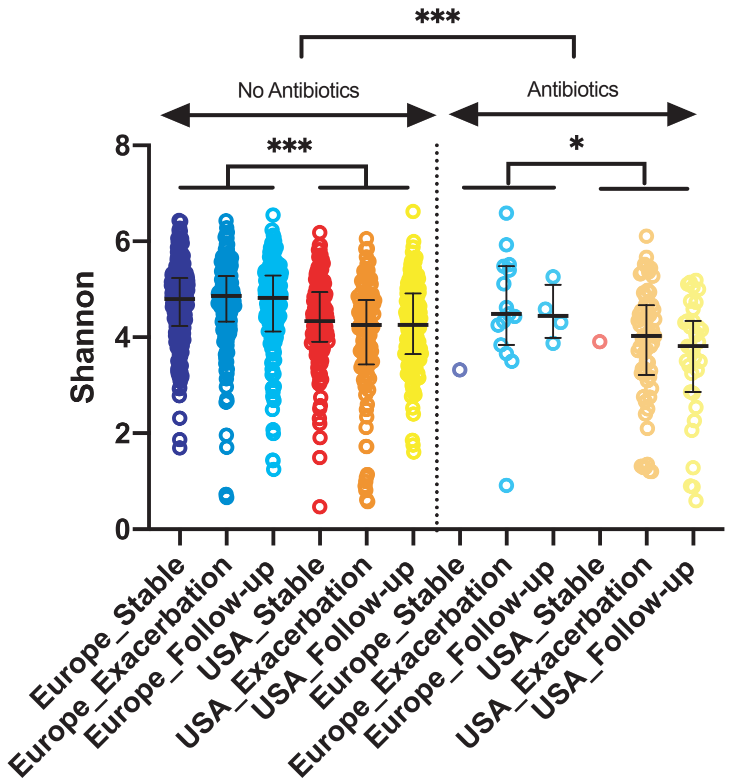

Supplementary Figure 1 – Shannon diversity following antibiotic treatment in Europe and USA patients. * *P*<0.05, *** *P*<0.0001

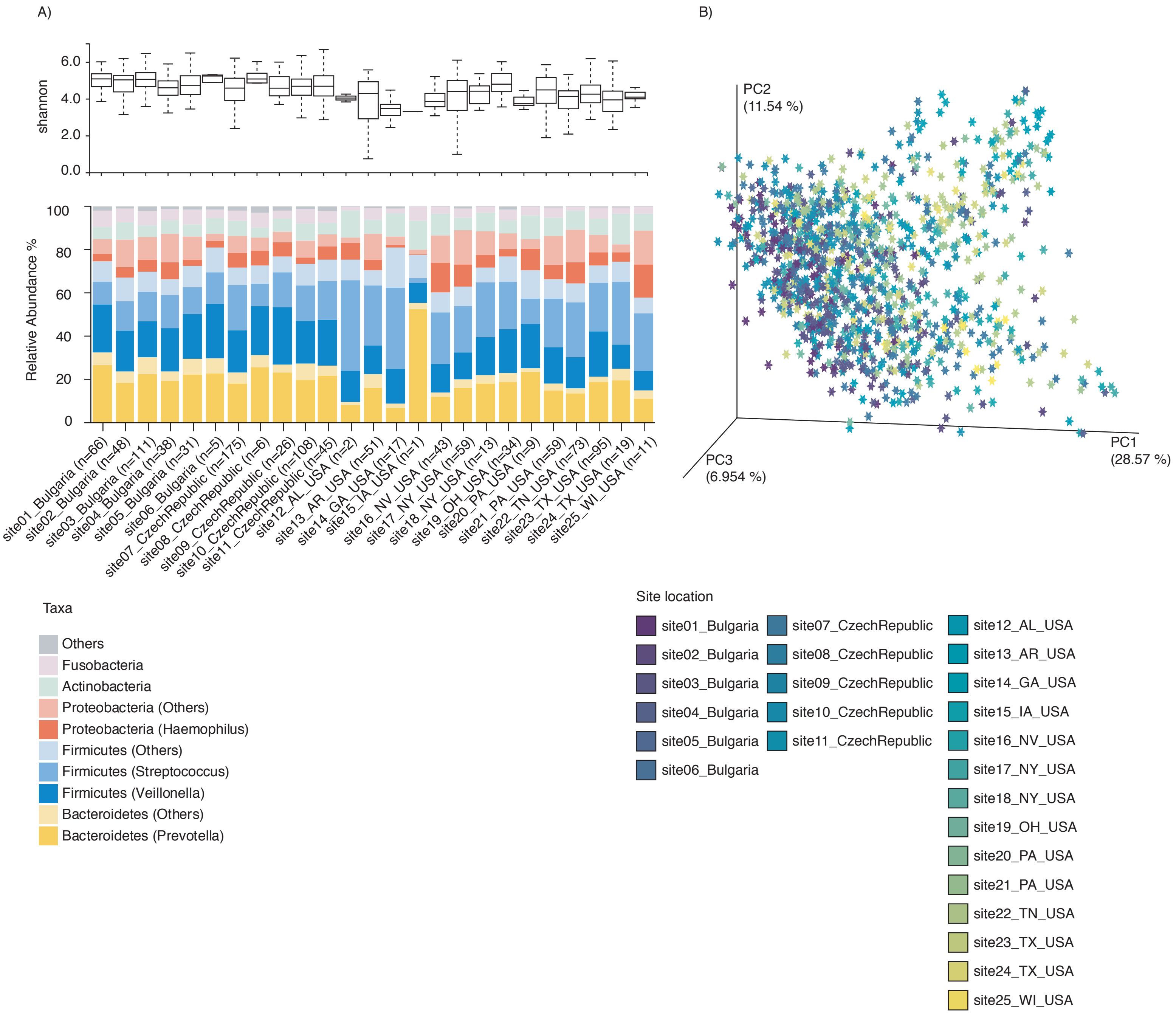

Supplementary Figure 2 – Microbiome composition and diversity across study sites. (A) Taxonomic barplot of major bacterial phyla and genera in samples grouped by geography and disease state, and their respective Shannon diversity index represented as boxplots with interquantile range whiskers. (B) Principal coordinate analysis of weighted Unifrac distances of the sputum microbiomes colored by sites

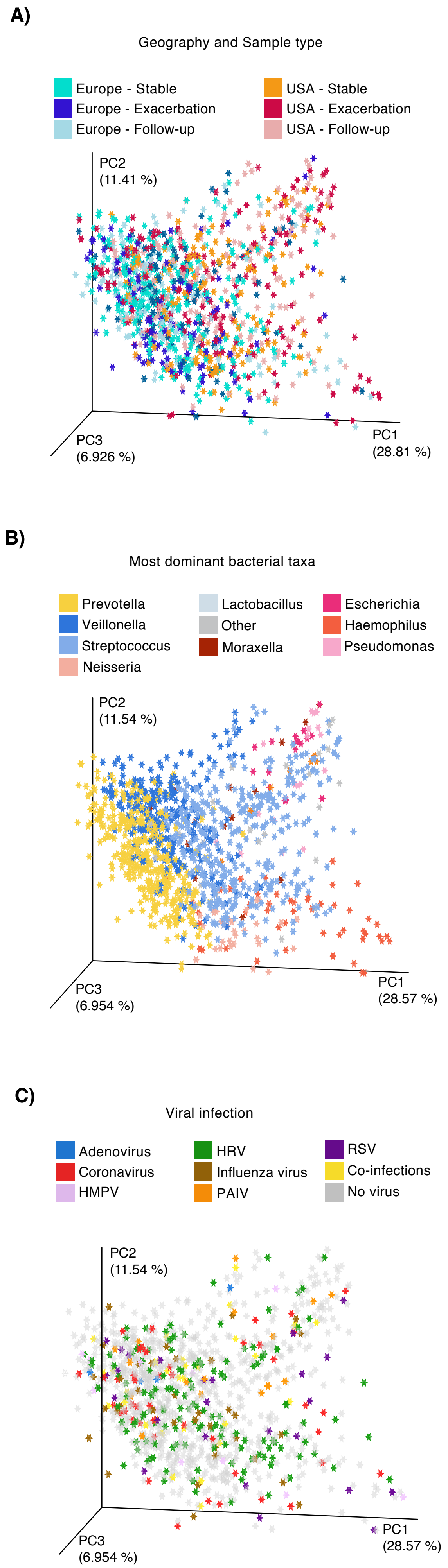

Supplementary Figure 3 – Principal coordinate analysis of weighted Unifrac distances of the sputum microbiomes colored by (A) geography and sample type, (B) most predominant bacterial taxa, and (C) viral infection

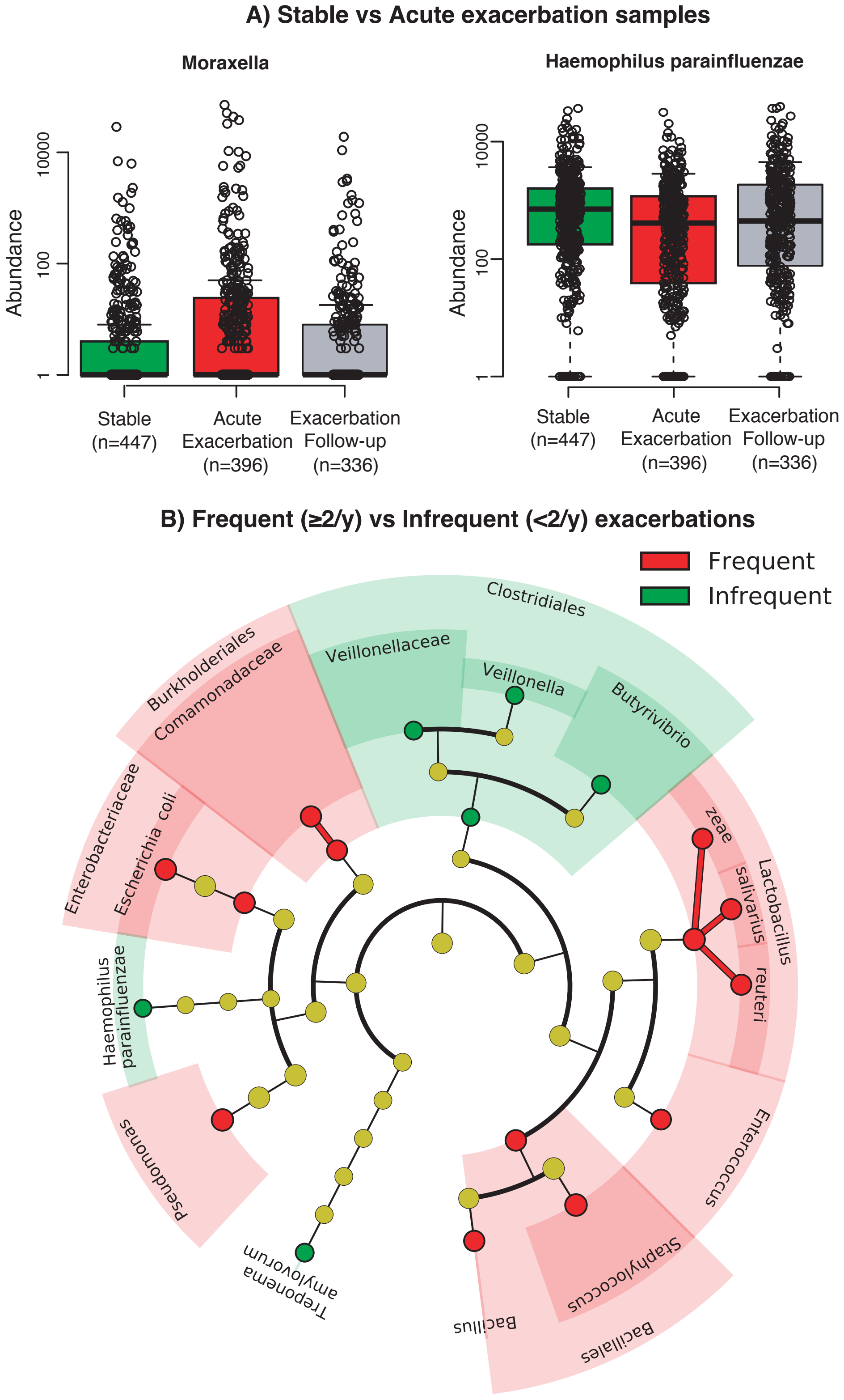

Supplementary Figure 4 – Bacteria associated with COPD exacerbation or frequency of exacerbation. (A) Abundance of Moraxella and *H.parainfluenzae* identified by analysis of composition (ANCOM) of microbiomes between stable and acute exacerbation samples. (B) Cladogram of bacterial taxa identified by ANCOM comparing Frequent (≥2 exacerbation event/ year) to Infrequent exacerbator

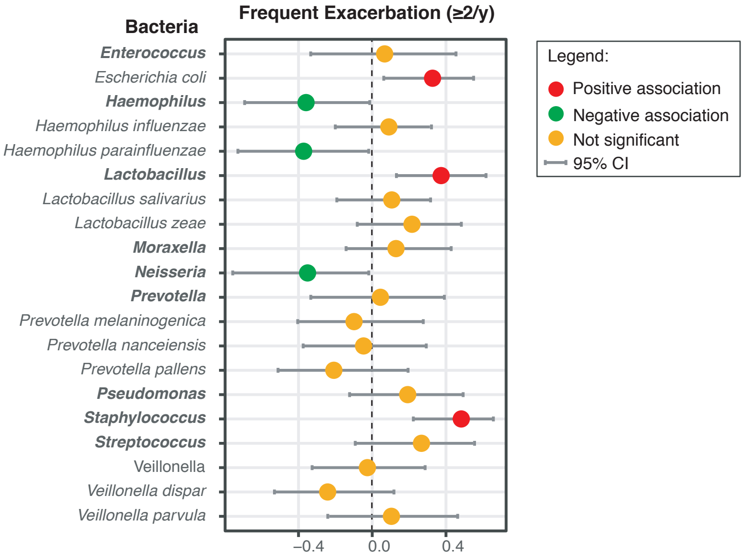

Supplementary Figure 5 – Adjusted odds ratio of bacterial abundance (top/bottom quartile) in stable samples only to be associated with frequent exacerbations.

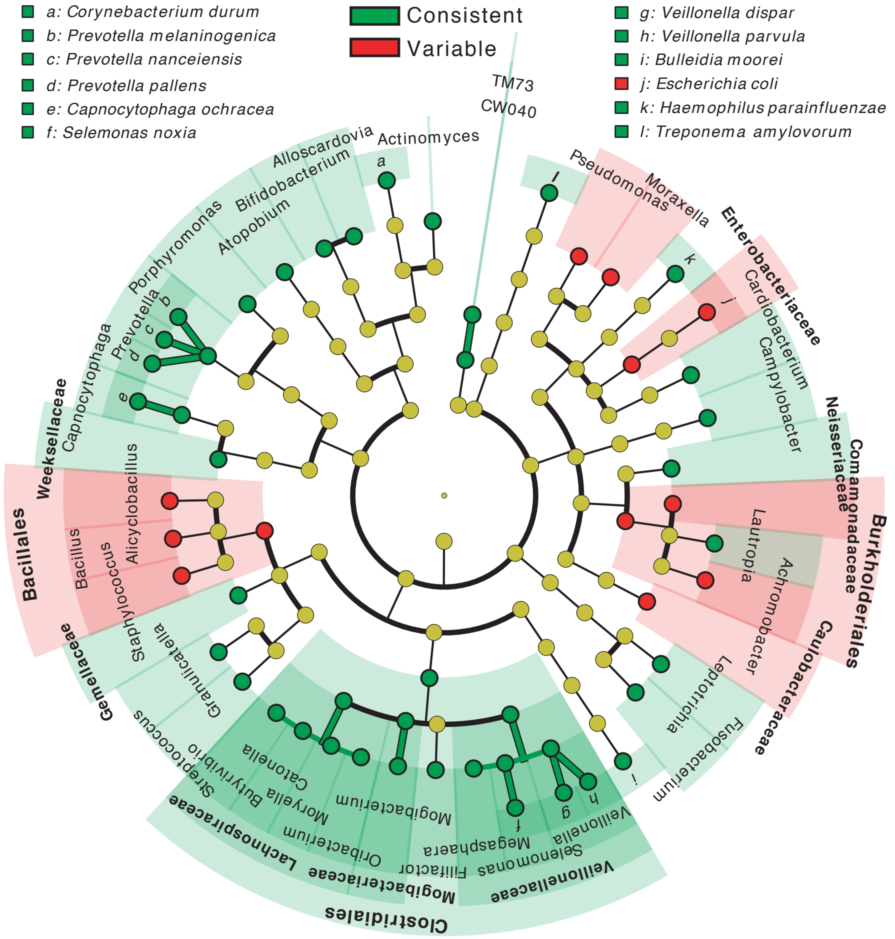

Supplementary Figure 6 – Cladogram of 55 Differentially abundant bacterial taxa identified by ANCOM comparing consistent to variable longitudinal microbiome patient profiles at stable state
